# Teacher or Nurse?

**Published:** 1921-03-05

**Authors:** 


*To the Editor of* The Hospital.

### Teacher or Nurse?

*To the Editor of* The Hospital.

Sir,—The recently proposed improvement in the salaries of female teachers in schools does not at present seem likely to materialise. A possible by-result of this disappointment is that some of these educational workers may turn their attention to the profession of nursing. Certainly some of them would be well qualified for the posts of sister-tutors now established in all the big hospitals. Also nursing would benefit in that its rank and file, being better educated and more accustomed to authority, would in time bring about a more democratic tradition in that profession. It is to be feared that as things are the matron is too much of an autocrat. Certainly the school teacher will find a larger and fuller field of usefulness in nursing. The Greeks were wont to surmise, if nothing had been heard of a man for some time, that he was better dead or a schoolmaster. Many amiable women have been buried, as it were, in their teaching sphere, which somehow conduces strongly to the celibacy of its votaries. Happily the same cannot be said of nursing, which, besides, is a calling intrinsically suited to the feminine genius.—Yours faithfully,

Caduceus.

January 24, 1921.

